# Photoelectric Properties of GaS_1−*x*_Se*_x_* (0 ≤ *x* ≤ 1) Layered Crystals

**DOI:** 10.3390/nano14080701

**Published:** 2024-04-18

**Authors:** Yu-Tai Shih, Der-Yuh Lin, Bo-Chang Tseng, Ting-Chen Huang, Yee-Mou Kao, Ming-Cheng Kao, Sheng-Beng Hwang

**Affiliations:** 1 Department of Physics, National Changhua University of Education, Changhua 500207, Taiwan; ytshih@cc.ncue.edu.tw (Y.-T.S.); ymkao@cc.ncue.edu.tw (Y.-M.K.); 2 Department of Electronic Engineering, National Changhua University of Education, Changhua 500208, Taiwan; 3 Graduate Institute of Photonics, National Changhua University of Education, Changhua 500207, Taiwan; 4 Department of Information and Communication Engineering, Chaoyang University of Technology, Taichung 413310, Taiwan; kmc@cyut.edu.tw; 5 Department of Electronic Engineering, Chienkuo Technology University, Changhua 500020, Taiwan; sbhwa@ctu.edu.tw

**Keywords:** photoelectric properties, GaS_1−*x*_Se*_x_* layered crystals, photoconductivity, Se composition, photocurrent, photoresponsivity

## Abstract

In this study, the photoelectric properties of a complete series of GaS_1−*x*_Se*_x_* (0 ≤ *x* ≤ 1) layered crystals are investigated. The photoconductivity spectra indicate a decreasing bandgap of GaS_1−*x*_Se*_x_* as the Se composition *x* increases. Time-resolved photocurrent measurements reveal a significant improvement in the response of GaS_1−*x*_Se*_x_* to light with increasing *x*. Frequency-dependent photocurrent measurements demonstrate that both pure GaS crystals and GaS_1−*x*_Se*_x_* ternary alloy crystals exhibit a rapid decrease in photocurrents with increasing illumination frequency. Crystals with lower *x* exhibit a faster decrease in photocurrent. However, pure GaSe crystal maintains its photocurrent significantly even at high frequencies. Measurements for laser-power-dependent photoresponsivity and bias-voltage-dependent photoresponsivity also indicate an increase in the photoresponsivity of GaS_1−*x*_Se*_x_* as *x* increases. Overall, the photoresponsive performance of GaS_1−*x*_Se*_x_* is enhanced with increasing *x*, and pure GaSe exhibits the best performance. This result contradicts the findings of previous reports. Additionally, the inverse trends between bandgap and photoresponsivity with increasing *x* suggest that GaS_1−*x*_Se*_x_*-based photodetectors could potentially offer a high response and wavelength-selectivity for UV and visible light detection. Thus, this work provides novel insights into the photoelectric characteristics of GaS_1−*x*_Se*_x_* layered crystals and highlights their potential for optoelectronic applications.

## 1. Introduction

GaS and GaSe are layered crystals belonging to the IIIA-VIA compound family and are classified as post-transition metal monochalcogenides [1,2,3]. The room-temperature bandgaps of GaS and GaSe reported in the literature range from 2.46 eV to 2.83 eV [4,5,6,7,8,9,10] and 1.95 eV to 2.03 eV [7,8,9,10,11,12,13,14], respectively. Therefore, GaS and GaSe have the potential to be used in the fabrication of optoelectronic devices for applications in the red and blue visible light regions. For instance, GaS has been described as a promising semiconductor for use in near-blue-light-emitting devices [15]. On the other hand, GaSe is also promising for optoelectronic devices in the visible range [16].

GaS and GaSe crystals can be fabricated into atomically thin, two-dimensional (2D) layered structures [17,18,19,20,21,22,23] due to the weak van der Waals forces between their adjacent monolayers [1,24]. Recent research has highlighted the intriguing properties exhibited by these 2D layered structures. For example, the photodetectors based on 2D layered structures of GaSe showed high performance of photoresponse in the UV and visible regions [19,22,25,26]. The GaSe thin film consisting of a few layers displayed a significant absorption coefficient [20]. Furthermore, strong second-harmonic generation was found in atomic layered GaSe [27,28]. On the other hand, the photodetectors based on 2D nanoflakes of GaS demonstrated a relevant UV-selective photoresponse [23]. The superior nonlinear optical activities were also found in the 2D layered structures of GaS [29]. The unique properties and high performance position 2D layered structures of GaS and GaSe as promising materials for applications in next-generation optoelectronics and electronics.

To engineer the properties of GaS and GaSe and expand their potential applications, alloying is one of the crucial methods. For instance, an entire series of GaS_1−*x*_Se*_x_* (0 ≤ *x* ≤ 1) alloy layered crystals can be produced [4,8,9,30,31,32,33,34,35,36,37]. By adjusting the composition ratio of S and Se, the lattice constants [9,30,32,33] and bandgap [8,9,36,37] can be tuned. The ability to tune the bandgap is essential for achieving high-performance optoelectronic devices. Numerous experimental and theoretical studies on GaS_1−*x*_Se*_x_* alloy crystals have been conducted [9,37,38,39,40,41,42,43,44], dating back to early reports on the photoconductivity of these alloys in 1960 [8]. For instance, the investigation on the nanobelts of GaS_1−*x*_Se*_x_* crystals showed they exhibited an intense photoluminescence spectrum in the visible range for all compositions [37]. More recently, GaS_1−*x*_Se*_x_* alloy crystals have been employed as color converters for GaN-based micro light-emitting diodes, enabling the conversion of blue emission to green and red [39]. The exploration of GaS_1−*x*_Se*_x_* alloy crystals for electronic and optoelectronic devices has garnered attention, yet comprehensive investigations into their photoelectric characteristics remain limited. Therefore, our study delves into the photoelectric properties of GaS_1−*x*_Se*_x_* alloy crystals, aiming to uncover their potential applications. Our experimental results reveal an enhancement in the photoresponsive performance of GaS_1−*x*_Se*_x_* with increasing *x*, with pure GaSe exhibiting the best performance. This finding contradicts previous observations on GaS_1−*x*_Se*_x_* nanobelts [37] and MoS_2(1−*x*)_Se_2*x*_ monolayers [45], where a decrease in photocurrents was noted with increasing Se composition *x*. Additionally, the inverse trends between the bandgap and photoresponsivity with increasing *x* suggests that the GaS_1−*x*_Se*_x_*-based photodetectors could potentially offer high response and specific wavelength-selectivity for UV and visible-light-detection applications by adjusting their composition and thickness. Thus, this work introduces novel insights and contributes to assessing the potential of GaS_1−*x*_Se*_x_* layered crystals for optoelectronic applications.

## 2. Materials and Methods

An entire series of GaS_1−*x*_Se*_x_* (0 ≤ *x* ≤ 1) layered crystals was grown using the chemical vapor transportation method with I_2_ as a transport agent. High-purity elemental S powder, Ga granules, Se granules, and I_2_ pieces were carefully weighed and placed in a quartz ampoule. The ampoule was then evacuated to 10^−6^ torr, sealed, and positioned in a three-zone furnace. To grow pure GaS crystals, the temperatures of the first, second, and third zones of the furnace were set to 950 °C, 900 °C, and 850 °C, respectively. For subsequent growths of GaS_1−*x*_Se*_x_* mixed crystals, the molar ratio *x* = Se/(S + Se) was incrementally increased by 0.2, accompanied by a corresponding reduction of 50 °C in the growth temperatures. Therefore, for the growth of pure GaSe crystals, the temperatures of the furnace’s first, second, and third zones were adjusted to 700 °C, 650 °C, and 600 °C, respectively. The crystal growth process typically lasted for approximately 265 h.

The scanning and transmission electron microscopy images of the grown GaS_1−*x*_Se*_x_* specimens revealed layered crystals with hexagonal structures and high crystalline quality. Raman and X-ray diffraction analyses indicated that GaS and GaS_0.8_Se_0.2_ were in the 2H β-phase, GaSe was in the 2H ε-phase, while those with intermediate *x* were in the 2H β-ε mixed phase. The wavelength of the samples’ photoluminescence peaks increased with *x* and covered the visible range. Detailed information regarding their growth conditions, composition ratios, structural properties, and optical properties has been previously reported [9]. This study focuses on investigating and reporting their photoelectric properties.

For the absorption and photoconductivity (PC) measurements, a 0.25 m monochromator (MKS, Irvine, CA, USA) equipped with a 130 W halogen lamp was employed to generate monochromatic light across a wide photon energy range. The continuous light from the monochromator was modulated into alternating light using a rotating beam chopper with a frequency set at 200 Hz, which then illuminated the measured sample. For the absorption measurements, a silicon photodetector (Thorlabs, Newton, NJ, USA) with a sensing range of 1.5 to 3.1 eV was positioned at the back of the measured sample. The output signals from the photodetector were captured using an EG&G 7265 Dual Phase DSP Lock-in Amplifier (Test Equipment Solutions, Bedfordshire, UK) to suppress noise signals effectively.

For the PC measurements, the sample under examination was affixed to a copper holder using thin tape. A stable bias voltage of 50 V was applied to the sample using a Keithley 2400 SourceMeter (Tektronix, Beaverton, OR, USA). The low power of the illuminating light, the exceptionally thin adhesive tape, and the efficient heat dissipation of the copper holder ensured minimal temperature rise in the sample during light exposure. This guaranteed that the current observed in the PC experiments stemmed from photoinduction rather than thermal induction. The signals of the induced photocurrent were received and transformed into voltage signals through an SR570 low-noise current preamplifier (Stanford Research Systems, Sunnyvale, CA, USA). An EG&G 7265 Dual Phase DSP Lock-in Amplifier processed the voltage signals, transferring them to a computer via a general-purpose interface bus (GPIB). The photoresponsivity of a measured sample was defined as the induced photocurrent divided by the power of the incident light. The variation in photoresponsivity with the photon energy of the incident light for a specimen was depicted as its PC spectrum.

To measure the photocurrent of a specimen as a function of time or the frequency of alternating illumination, a laser with a wavelength of 405 nm was employed as the excitation source. This laser, controlled by an AFG-2225 function generator (GW Instek, New Taipei City, Taiwan), applied on/off light modulation to the measured specimen. A stable bias voltage of 50 V was applied to the sample using a Keithley 2400 SourceMeter. An SR570 low-noise current preamplifier received the signals of the induced photocurrent and transformed them into voltage signals. For the time-dependent photocurrent measurements, a data acquisition device with a time resolution of 1 μs was employed to collect and transfer these signals to a computer for depicting the variation of photocurrent over time for the measured specimen. For the frequency-dependent photocurrent measurements, an EG&G 7265 Dual Phase DSP Lock-in Amplifier was used to receive the voltage signals and transfer them to a computer via a GPIB. The amplitude of the alternating photocurrent during alternating illumination, *I*_ac_, was divided by the steady-state photocurrent during steady illumination, *I*_dc_, to obtain the normalized photocurrent *I*_ac_/*I*_dc_ as a function of the frequency of alternating illumination. 

To measure the photoresponsivity of a specimen as a function of the incident laser power or the bias voltage, a laser with a wavelength of 405 nm was employed as the excitation source. An AFG-2225 function generator was utilized to modulate the laser light into alternating light with a frequency of 1 Hz. For the bias-dependent photoresponsivity measurements, the laser power was set to 11.6 mW, and a Keithley 2400 SourceMeter was used to apply a bias voltage to the measured sample and record the induced current. The difference between the average currents under illumination and in the dark was divided by the incident laser power to obtain the photoresponsivity of the measured sample. For the laser-power-dependent photoresponsivity measurements, the incident laser power was adjusted using neutral-density filters. A stable bias voltage of 50 V was applied to the measured sample using a Keithley 2400 SourceMeter. The photocurrent was recorded using an EG&G 7265 Dual Phase DSP Lock-in Amplifier and then divided by the incident laser power to obtain the photoresponsivity of the measured sample.

## 3. Results and Discussion

**Figure 1:**
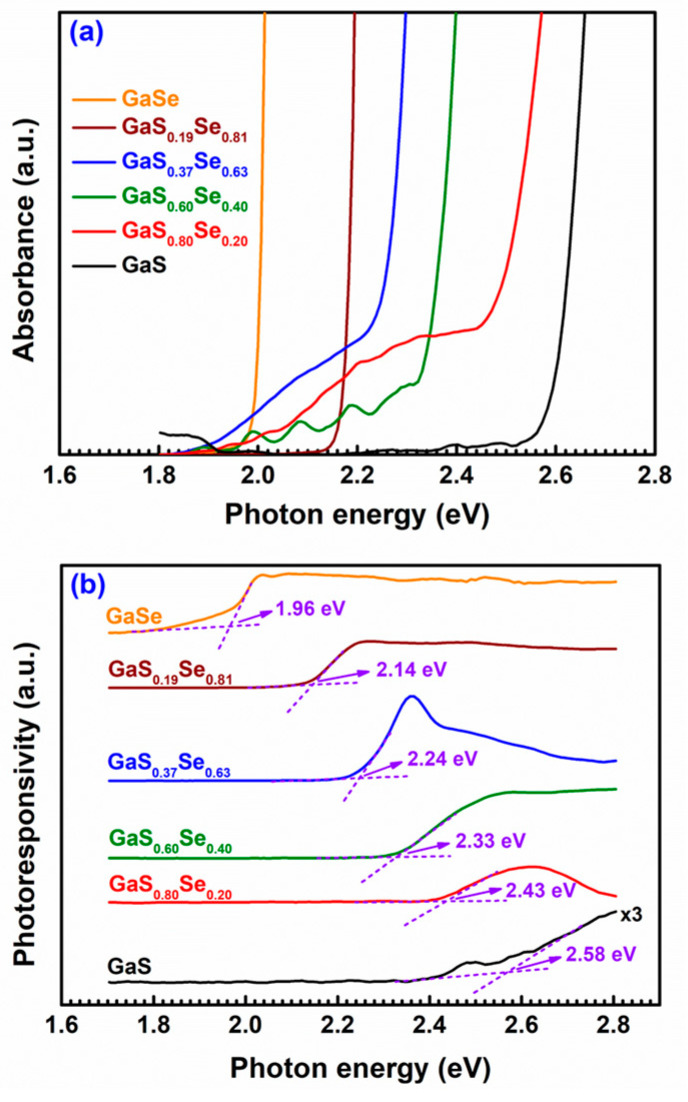
Absorption (**a**) and photoconductivity (**b**) spectra of the GaS_1−*x*_Se*_x_* specimens at room temperature.

The room-temperature absorption spectra of the GaS_1−*x*_Se*_x_* (0 ≤ *x* ≤ 1) samples are depicted in Figure 1a. Based on these absorption spectra, we employed the Tauc plot method [46,47] to ascertain the indirect and direct bandgaps of the GaS_1−*x*_Se*_x_* samples by extrapolating the linear segment of the (*AE*_ph_)*^n^* vs. *E*_ph_ curves at (*AE*_ph_)*^n^* = 0 for *n* = 1/2 and 2, respectively. Here, *A* represents the absorbance of the measured sample, and *E_ph_* signifies the energy of the incident photon. The determined indirect bandgaps of GaS, GaS_0.80_Se_0.20_, GaS_0.60_Se_0.40_, GaS_0.37_Se_0.63_, GaS_0.19_Se_0.81_, and GaSe are 2.58 eV, 2.45 eV, 2.34 eV, 2.24 eV, 2.16 eV, and 2.00 eV, respectively. Similarly, the determined direct bandgaps of GaS, GaS_0.80_Se_0.20_, GaS_0.60_Se_0.40_, GaS_0.37_Se_0.63_, GaS_0.19_Se_0.81_, and GaSe are 2.64 eV, 2.49 eV, 2.36 eV, 2.28 eV, 2.18 eV, and 2.02 eV, respectively [9]. It is noted that, as *x* increases, the sample exhibits a smaller bandgap.

The room-temperature PC spectra of the GaS_1−x_Se*_x_* (0 ≤ *x* ≤ 1) samples are illustrated in Figure 1b. It is apparent that the photoconductivity of each specimen undergoes negligible variations with increasing photon energy until it experiences a sudden increase beyond a specific value. This specific value roughly indicates the bandgap of the specimen. When the photon energy surpasses the bandgap of the semiconductor material, electrons can absorb photons and transition from the valence band to the conduction band, resulting in an increase in the total number of conduction carriers and subsequently enhancing the material’s conductivity. The bandgaps of GaS, GaS_0.80_Se_0.20_, GaS_0.60_Se_0.40_, GaS_0.37_Se_0.63_, GaS_0.19_Se_0.81_, and GaSe are approximately 2.58 eV, 2.43 eV, 2.33 eV, 2.24 eV, 2.14 eV, and 1.96 eV, respectively. The bandgaps determined from the PC spectra of the GaS_1−*x*_Se*_x_* samples align with those indirect bandgaps obtained from their absorption spectra.

**Figure 2:**
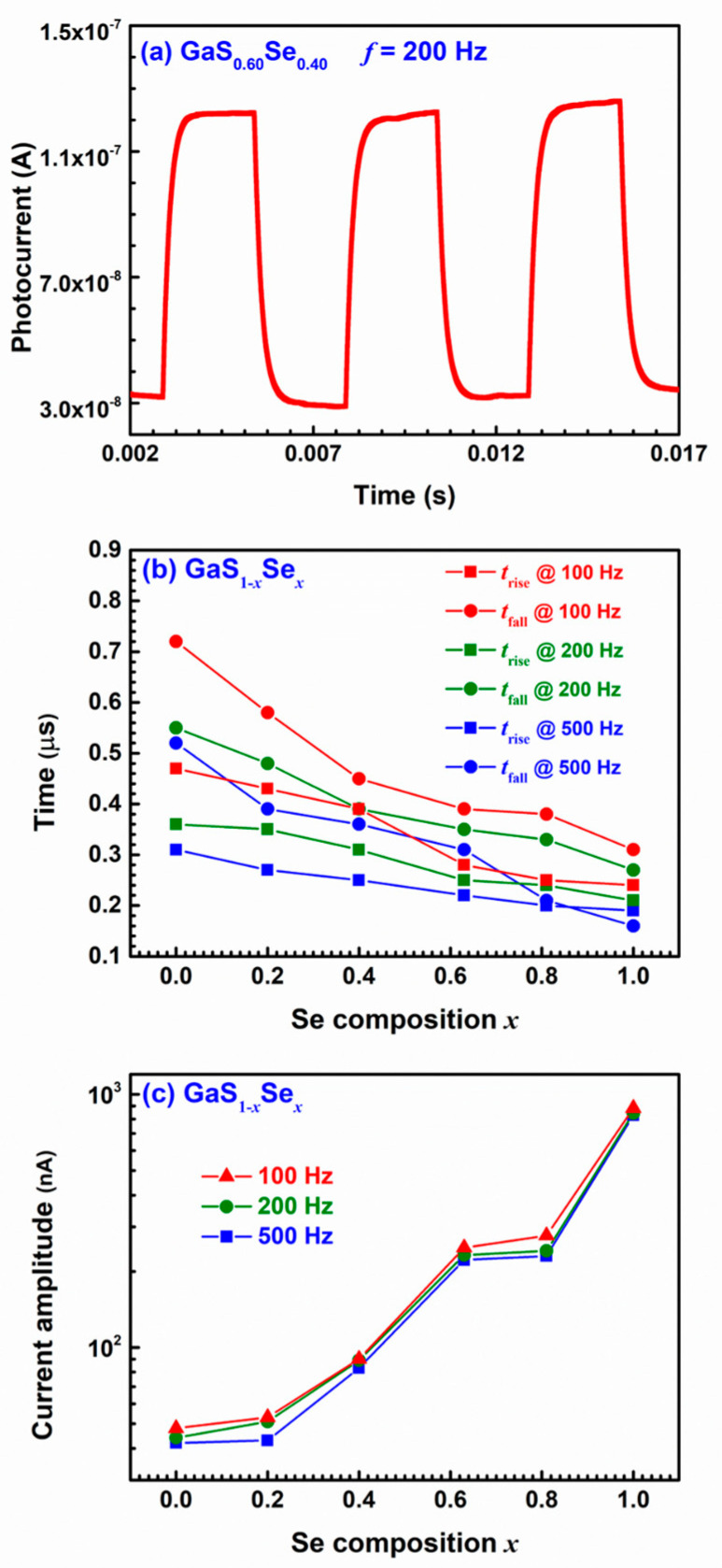
(**a**) Photocurrent of the GaS_0.60_Se_0.40_ specimen under an illumination frequency of 200 Hz as a function of time. Rise times *t*_rise_ and fall times *t*_fall_ (**b**) and current amplitudes (**c**) of the GaS_1−*x*_Se*_x_* specimens under variant frequencies of alternating illumination as functions of the Se composition *x*.

Figure 2a presents the photocurrent profile of the GaS_0.60_Se_0.40_ specimen under an illumination frequency of 200 Hz, depicting its variation over time. Similar behaviors were observed for the photocurrents of other GaS_1−*x*_Se*_x_* specimens under different illumination frequencies. Figure 2b illustrates the rise times *t*_rise_, defined from 10% to 90% of the maximum photocurrent, and the fall times *t*_fall_, defined from 90% to 10% of the maximum photocurrent, for the GaS_1−*x*_Se*_x_* samples under various illumination frequencies as functions of the Se composition *x*. Across all illumination frequencies, *t*_rise_ and *t*_fall_ decrease with an increase in the Se composition *x*. The GaSe sample exhibits the shortest *t*_rise_ and *t*_fall_.

In Figure 2c, the current amplitudes, representing the difference between the maximum and minimum photocurrents in a rising–falling period, are shown for the GaS_1−*x*_Se*_x_* samples under different illumination frequencies. Regardless of the illumination frequency, the current amplitude rises with an increase in the Se composition *x*. The pure GaSe sample attains the highest current amplitude, significantly surpassing those of the GaS_1−*x*_Se*_x_* ternary alloy samples and the pure GaS sample. This finding contrasts with previous investigations on both GaS_1−*x*_Se*_x_* nanobelts [37] and MoS_2(1−*x*)_Se_2*x*_ monolayers [45], which indicated a decrease in photocurrent as the Se composition *x* increased. Our result demonstrates the opposite trend.

**Figure 3:**
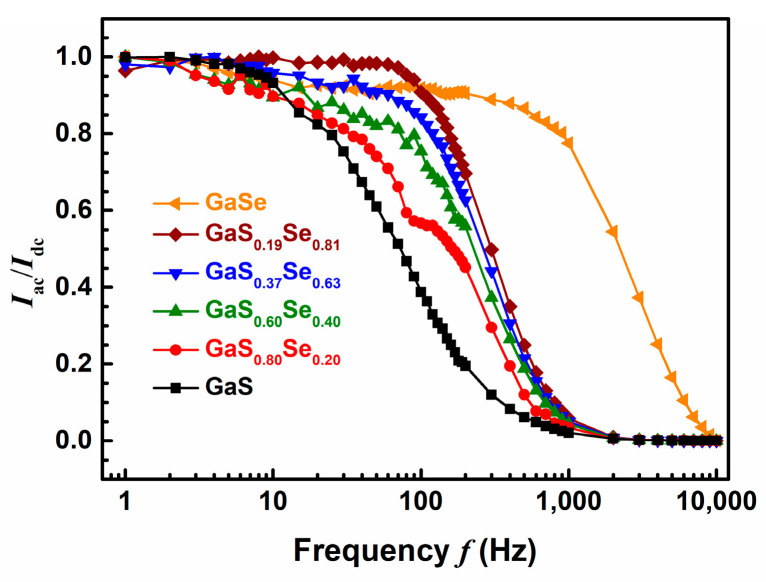
Normalized photocurrents of the GaS_1−*x*_Se*_x_* specimens as functions of the frequency of alternating illumination.

Figure 3 illustrates the normalized photocurrents *I*_ac_/*I*_dc_ of the GaS_1−*x*_Se*_x_* specimens as functions of the frequency *f* of alternating illumination. For frequencies *f* ≥ 100 Hz, a higher Se composition *x* corresponds to a greater *I*_ac_/*I*_dc_. The normalized photocurrents decrease rapidly with increasing *f* for the pure GaS sample and the GaS_1−*x*_Se*_x_* ternary alloy samples, with the photocurrent dropping more quickly for samples with lower *x*. For frequencies above 1000 Hz, the *I*_ac_/*I*_dc_ for these samples are less than 0.01. In contrast, the normalized photocurrent for GaSe remains significantly high even at high frequencies, exceeding 0.01 at *f =* 9000 Hz, with an *I*_ac_/*I*_dc_ greater than 0.015. Consequently, at high frequencies of alternating illumination, the optical response of the pure GaSe sample surpasses that of both the pure GaS sample and the GaS_1−*x*_Se*_x_* ternary alloy samples.

**Figure 4:**
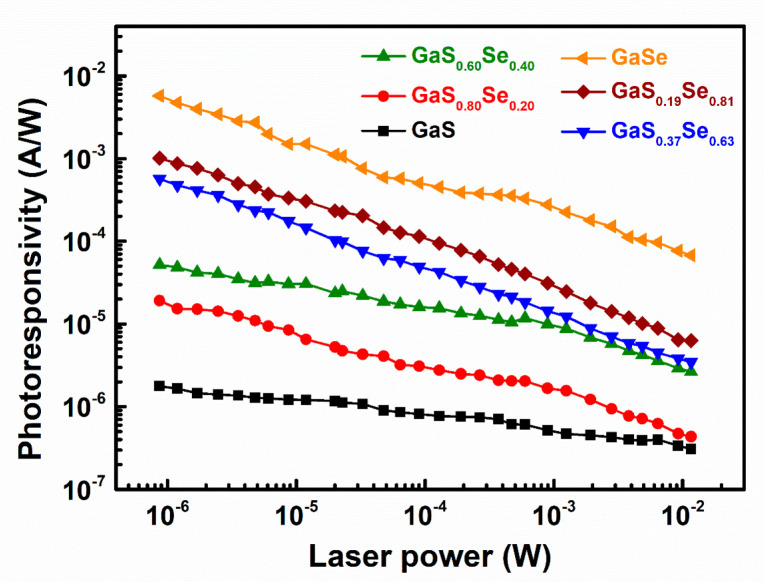
Photoresponsivities of the GaS_1−*x*_Se*_x_* specimens as functions of the incident laser power.

Figure 4 illustrates the photoresponsivities of the GaS_1−*x*_Se*_x_* specimens as functions of the incident laser power. As the incident laser power gradually decreases from the order of 10^−2^ W to the order of 10^−6^ W, the photoresponsivities of all GaS_1−*x*_Se*_x_* samples gradually increase. The GaSe sample exhibits the largest increase, with its photoresponsivity increasing by 85.7 times. For a given incident laser power, the photoresponsivity increases as *x* increases. The pure GaSe sample possesses the highest photoresponsivity at any laser power, exceeding that of other samples by at least one order of magnitude. The maximum photoresponsivity of the pure GaSe sample reaches 5.77 × 10^−3^ A/W at a laser power of 8.70 × 10^−7^ W, significantly greater than those of the GaS_1−*x*_Se*_x_* ternary alloy samples and the pure GaS sample. Conversely, the pure GaS sample has the lowest maximum photoresponsivity at a laser power of 8.70 × 10^−7^ W, which is 1.78 × 10^−6^ A/W. 

**Figure 5:**
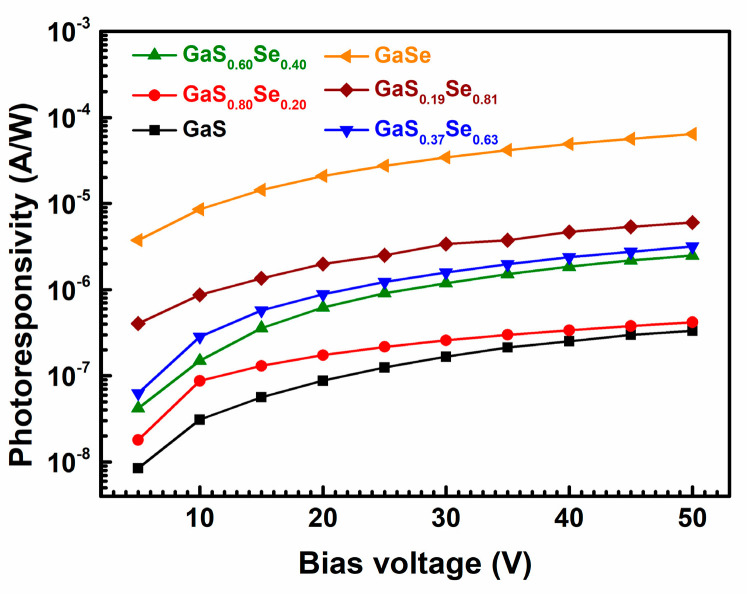
Photoresponsivities of the GaS_1−*x*_Se*_x_* specimens as functions of the bias voltage.

Figure 5 illustrates how the photoresponsivities of the GaS_1−*x*_Se*_x_* samples vary with the bias voltage. As the applied bias voltage increases from 5 V to 50 V, the photoresponsivities of all GaS_1−*x*_Se*_x_* samples gradually increase. For a given bias voltage, the photoresponsivity increases as *x* increases. The pure GaSe sample exhibits the highest photoresponsivity at any bias voltage, exceeding that of other samples by at least one order of magnitude. At a bias voltage of 5 V, the photoresponsivities of the GaS_1−*x*_Se*_x_* samples increase from 8.44 × 10^−9^ A/W to 3.76 × 10^−6^ A/W as *x* increases from 0 to 1. This contrasts with the findings of Jung et al. [37], who reported that the photoresponsivities of GaS_1−*x*_Se*_x_* nanobelts at 2 V were approximately 7 × 10^−6^ A/W for *x* ranging from 0 to 0.5, decreasing to approximately 1 × 10^−6^ A/W for *x =* 0.7 and approximately 0.5 × 10^−6^ A/W for *x =* 1. Our measured photoresponsivity changes with *x* in the opposite direction of theirs. The photoresponsivity of our pure GaSe sample reaches its maximum value of 6.39 × 10^−5^ A/W at 50 V, significantly greater than those of the GaS_1−*x*_Se*_x_* ternary alloy samples and the pure GaS sample. Conversely, the pure GaS sample has the lowest maximum photoresponsivity at a bias voltage of 50 V, which is 3.34 × 10^−7^ A/W.

Ho et al. investigated the photoconductance and photoresponse of the photodetectors based on GaS_1−*x*_Se*_x_* layered crystals, ranking the photosensitivity of their samples from maximum to minimum as GaS_0.3_Se_0.7_, GaS_0.2_Se_0.8_, GaS_0.4_Se_0.6_, GaS_0.1_Se_0.9_, GaSe, and GaS [48]. However, our results, as discussed above, differ from those of Ho et al. [48]. We found that the photoresponsive performance of the GaS_1−*x*_Se*_x_* samples improves monotonously with increasing *x*, with the pure GaSe sample exhibiting the best performance.

The enhancement in photoresponsive performance may be attributed to the decreased bandgaps of the GaS_1−*x*_Se*_x_* samples with increasing *x*. The redshift of the bandgaps occurs because an increase in the Se composition *x* results in more S ions being replaced by Se ions, leading to an increase in lattice constants and, consequently, a decrease in bandgaps. A smaller bandgap facilitates the transition of an electron from the valence band to the conduction band by absorbing a photon. The increased number of carriers generated by the absorption of photons enhances the photoresponsive performance of the semiconductors.

Another factor contributing to enhancing the photoresponsive performance of GaS_1−*x*_Se*_x_* layered crystals may be the reduction in the difference between indirect and direct bandgaps as *x* increases. The minimum difference is only 20 meV when *x =* 1. This insignificant energy difference makes the GaS_1−*x*_Se*_x_* crystals with high *x* resemble pseudo-direct bandgap semiconductors [9]. Semiconductors with direct bandgaps exhibit better response to light. Consequently, the photoresponsive performance of the GaS_1−*x*_Se*_x_* crystals is enhanced with increasing *x*. 

It is well known that GaX (X = S or Se) layered crystals exhibit four basic polytypes determined by the stacking sequences of the monolayers: 2H β-, 2H ε-, 3R γ-, and 4H δ-GaX, corresponding to the space groups *P*6_3_/*mmc* (*D*_6*h*_^4^), $P\bar{6}m2$(*D*_3*h*_^1^), *R*3*m* (*C*_3*v*_^5^), and *P*6_3_*mc* (*C*_6*v*_^4^) [9]. These distinct stacking arrangements give rise to variations in the properties of GaX crystals across different phases. Building upon this understanding, we propose another intriguing hypothesis suggesting that the photoresponsive performance of GaS_1−*x*_Se*_x_* layered crystals may be influenced by their polytypes. Jung et al. [37] demonstrated that their pure GaS existed in the β phase, while the GaS_1−*x*_Se*_x_* ternary alloys and pure GaSe were in a β-γ mixed phase. With increasing *x*, the ratio of β to γ phases decreased. Conversely, our pure GaS and GaS_0.80_Se_0.20_ ternary alloy were in the β-phase. GaS_0.60_Se_0.40_, GaS_0.37_Se_0.63_, and GaS_0.19_Se_0.81_ ternary alloys exhibited a β-ε mixed phase, with the ratio of β to ε phases decreasing with increasing *x*. Pure GaSe was in the ε phase. If we hypothesize that the ε and γ phases have the best and worst photoresponsive performance, respectively, with the β phase exhibiting intermediate performance, it may explain the contrast between our findings and those of Jung et al. [37], who reported a decrease in the photoresponsive performance of GaS_1−*x*_Se*_x_* nanobelts with increasing *x*. However, growing GaS_1−*x*_Se*_x_* crystals exclusively in one pure phase for all different phases remains challenging. Therefore, confirming this hypothesis would require the development of a state-of-the-art growth technique for GaS_1−*x*_Se*_x_* crystals, presenting an intriguing task for future research.

The bandgaps of GaS_1−*x*_Se*_x_* layered crystals span the visible region, but they can significantly increase to over 3.0 eV and extend into the UV region when reduced to the monolayer state due to the quantum confinement effect [36,49]. Photodetectors based on 2D nanoflakes of GaS have shown a remarkable UV-selective photoresponse attributed to the increased bandgaps [23,50,51]. Considering the photoelectric properties of GaS_1−*x*_Se*_x_* layered crystals, the decrease in bandgaps with increasing *x* implies that GaS_1−*x*_Se*_x_* crystals with *x* > 0 respond to longer wavelengths compared to pure GaS crystals. Additionally, the enhancement of photoresponsive performance with increasing *x* suggests that GaS_1−*x*_Se*_x_* crystals with *x* > 0 exhibit higher photoresponsivity than pure GaS crystals. Consequently, GaS_1−*x*_Se*_x_*-based photodetectors could potentially offer high response and specific wavelength-selectivity for UV and visible-light-detection applications by adjusting their composition and thickness, thereby broadening the potential applications of GaS_1−*x*_Se*_x_* layered crystals in optoelectronics.

## 4. Conclusions

The photoelectric properties of a complete series of GaS_1−*x*_Se*_x_* (0 ≤ *x* ≤ 1) layered crystals were investigated. The photoconductivity spectra revealed that the bandgap of GaS_1−*x*_Se*_x_* gradually decreases as the Se composition *x* increases. The time-dependent photocurrent measurements demonstrated that the increase in *x* significantly improves the response of GaS_1−*x*_Se*_x_* to light. The pure GaSe crystal has the shortest rise and fall times and the largest current amplitude. The frequency-dependent photocurrent measurements indicated that the photocurrents of the pure GaS crystal and GaS_1−*x*_Se*_x_* ternary alloy crystals decrease rapidly as the frequency of alternating illumination increases. Crystals with lower *x* have a faster decrease in photocurrent. However, the photocurrent of the pure GaSe crystal persists significantly, even at high frequencies. Additionally, the measurements for laser-power-dependent photoresponsivity and bias-voltage-dependent photoresponsivity revealed that the increase in *x* enhances the photoresponsivity of GaS_1−*x*_Se*_x_*. Overall, the photoresponsive performance of GaS_1−*x*_Se*_x_* improves with increasing *x*, with pure GaSe exhibiting the best performance. This result contradicts the findings of the previous report. This enhancement in photoresponsive performance of the GaS_1−*x*_Se*_x_* layered crystals may be attributed to several factors, including decreasing bandgaps, reduced differences between indirect and direct bandgaps, and phase conversion from β-GaS to ε-GaSe as *x* increases. Because the bandgap and the photoresponsivity of GaS_1−*x*_Se*_x_* vary in reverse trends as *x* increases, the GaS_1−*x*_Se*_x_*-based photodetectors could potentially offer high response and specific wavelength-selectivity for UV- and visible-light-detection applications by adjusting their composition and thickness. The findings of this work provide novel insights into the photoelectric characteristics of GaS_1−*x*_Se*_x_* layered crystals and contribute to assessing the viability of GaS_1−*x*_Se*_x_* layered crystals for potential use in optoelectronics.

## Data Availability

Data are contained within the article.
